# Challenging the knowledge base and skillset for providing surgical consent by orthopedic and plastic surgeons in the Netherlands: an identified area of improvement in patient safety

**DOI:** 10.1186/s13037-016-0110-0

**Published:** 2016-10-22

**Authors:** Wouter K. G. Leclercq, Sarah Sloot, Bram J. Keulers, Saskia Houterman, Johan Legemaate, Margot Veerman, Leslie Thomas, Marc R. Scheltinga

**Affiliations:** 1 Department of Surgery, Máxima Medical Centre, De Run 4600, 5504 DB Veldhoven, The Netherlands; 2 Department of Surgery, UMCG, Groningen, The Netherlands; 3 Department of Plastic Surgery, Bernhoven Hospital, Uden, The Netherlands; 4 Department of Education and Research, Catharina Hospital, Eindhoven, The Netherlands; 5 Department of Public Health, Academic Medical Centre, University of Amsterdam, Amsterdam, The Netherlands; 6 Department of plastic Surgery, Isala Hospital, Zwolle, The Netherlands; 7 School of Information, University of South Florida, Tampa, USA

**Keywords:** Informed consent, Orthopedic surgery, Plastic surgery, Preoperative care, Surgery

## Abstract

**Background:**

Successfully completing a surgical informed consent process is an important element of the preoperative consult. A previous study of Dutch general surgeons demonstrated that the implementation of SIC did not meet acceptable standards. However, the quality of the SIC process in the orthopedic surgical or plastic surgical arena is unknown.

**Methods:**

Following ethical approval, an online survey investigating specifics of surgical informed consent was performed among members of the Dutch Scientific Association of Orthopedic Surgeons and the Dutch Society for Plastic Surgery.

**Results:**

A total of 335 responses from a majority of departments of orthopedic (86 %) and plastic surgery (78 %) were eligible for analysis. Scores on knowledge were poor as only 50 % recognized the three basic elements of surgical informed consent (competence, exchange of information and consent). The orthopedic group used more tools in the surgical informed consent process, such as instruction movies and websites or specialized nursing staff, compared to plastic surgery (orthopedic: 31-50 % vs. plastic: 6-30 %, *p* = 0.05- < 0.001). In contrast, surgical informed consent forms were used more frequently by the plastic surgical group (orthopedic 21 % vs. plastic:42 % *p* < 0.001). Control of the efficacy of the surgical informed consent process was low, 36 % in both groups. One in every seven orthopedic or plastic surgeons was faced with an official surgical informed consent-related complaint in the previous five years.

**Conclusions:**

Similar to general surgeons, Dutch orthopedic and plastic surgeons demonstrate poor knowledge and skills regarding surgical informed consent. Increased awareness, better training and use of modern tools including standard forms and online software programs will improve the SIC process and will optimize patient care.

**Electronic supplementary material:**

The online version of this article (doi:10.1186/s13037-016-0110-0) contains supplementary material, which is available to authorized users.

## Background

Each patient is entitled to detailed information prior to an invasive or a surgical procedure. Once properly educated by a surgeon, a shared decision is made whether or not to perform the proposed operation. These basic rights and obligations are incorporated into the surgical informed consent (SIC) process [1–4]. However, daily practice is troublesome as just a minority of patients are offered the opportunity to complete all stages of this SIC process [3, 5, 6]. General goals of SIC are to anchor the patient’s authority and to enhance safety, satisfaction and compliance [3, 5, 6]. If the quality of the SIC process is high, patients are better informed and more compliant while results after surgery may be optimized [3, 5, 6]. Moreover, patient expectations are more realistic, resulting in more trust in their surgeon and/or surgical procedure and eliciting fewer complaints [3, 5, 6].

Since 1995, the Dutch Medical Treatment Contract Act (WGBO) has provided legal requirements on informed consent in the Netherlands. This act describes the legal boundaries of SIC in a civil law approach – in other words, in a contract that is made between a healthcare provider and a patient [7]. In contrast, other countries may have explored alternative views. For instance, legislation in Scandinavia is based upon a public approach formulating ‘obligations imposed on physicians and other healthcare providers’. Therefore, a private contract between one doctor and one patient is not required in these countries [7].

**Fig. 1:**
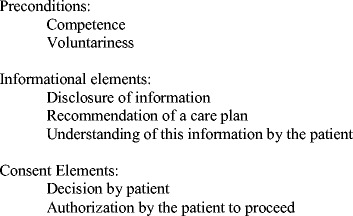
Elements of informed consent

Although details of legislation differ per country, the three elements of SIC are equal in western countries. Features of consecutive steps of a correctly executed SIC process were previously reported (Fig. 1) [3, 8]. Firstly, it should be checked whether patients are competent and free (without pressure) to decide. Secondly, information on diagnosis, prognosis, procedures, benefits, risks and alternative strategies including postponing surgery should be provided. A check on whether the information is understood is also required. The final stage includes adequate recording. Written consent for invasive procedures is strongly recommended, although a patient’s (or representative’s) signature is not obliged in many European countries, including the Netherlands [7].

A recent survey investigating daily practice regarding SIC demonstrated that knowledge of general surgeons (GS) in the Netherlands was inadequate and guidelines were often not followed [9]. Only 55 % of surgeons were familiar with the three basic elements of SIC. Daily practice varied widely between surgeons and residents. This practice resulted in 17 % of the surgeons facing an SIC-related complaint in the previous five years. Several other Dutch surgical organizations, including the Scientific Association of Orthopedic Surgeons and the Dutch Society for Plastic Surgery, have also adopted guidelines on SIC [10, 11]. However, it is unknown whether these Dutch specialists perform any better regarding these issues compared to their fellow general surgeons.

The general aim of this study was to analyze knowledge and daily practice concerning SIC in orthopedic surgeons (OS) and plastic surgeons (PS). As OS and PS procedures are mainly elective and frequently based upon functional or cosmetic complaints, it was hypothesized that both of these subspecialists would score comparably regarding knowledge and skills in SIC. Moreover, both groups were expected to have more knowledge and a better implementation in daily practice when compared with general surgeons. Possible differences between surgeons and their residents were also studied.

## Methods

The study was performed between January 2011 and December 2011 in Máxima Medical Centre, a large teaching hospital in the southern part of the Netherlands. Orthopedic surgical procedures were performed in all 94 public Dutch hospitals (8 university, 26 large teaching and 60 general hospitals). Plastic surgery is primarily offered in larger hospitals (approximately 50/94). Orthopedic and plastic surgical care are also provided on a much smaller scale in a limited number of private clinics serving as day care facilities.

Details of the survey were published previously [9]. In summary, an existing questionnaire that was used in a survey among general surgeons was modified and optimized by an OS (JvM) and a PS consultant (HW) (Additional file 1). All questions were multiple choice. General characteristics, knowledge and daily practice were asked. Endorsed and facilitated by both the Scientific Association of Orthopedic Surgeons (NOV) and the Dutch Society for Plastic Surgery (NVPC), an e-mail linked to an online multiple-choice questionnaire was sent in February 2011 and October 2011 to all actively practicing OS and PS surgeons and residents in the Netherlands (Table 1). A reminder was sent to non-responders one month later, shortly followed by an official study closure towards the end of 2011. Participants were excluded from analysis if they did not recently work in the Netherlands.

**Table 1:** Population and response

	Total number	Response	Percent
Total	1177	335	28.5
Surgeons	879	265	30.1
Residents	298	70	23.5
OS total	843	253	30.0
Surgeons	624	206	33.0
Residents	219	45	20.5
PS total	334	84	25.1
Surgeons	255	59	23.1
Residents	79	25	31.6

*OS* Orthopedic Surgery, *PS* Plastic Surgery

### Ethics, consent and permissions

Ethical approval was obtained from the Institutional Review Board of Máxima Medical Centre.

### Statistical analysis

All data were collected in an online database, checked for duplicates, and immediately rendered anonymous. Descriptive statistics were used to analyze the data. *χ* ^2^ or Fisher’s Exact tests (in the case of small numbers) were used to compare OS with PS surgeons and surgeons with residents. A *p* < 0.05 was considered significant. Statistical analyses were performed using version 18 SPSS, Chicago, Illinois, USA.

## Results

### Representativeness of the study

Responses were received from 81 of the 94 OS departments (86 %) and 39 of the 50 PS departments (78 %). 335 individual answers (28 %, 335 of 1177) from surgeons (S, *n* = 267) and residents (R, *n* = 70) were eligible for analysis (Table 1). General characteristics of both groups are presented in Table 2. Of course, age and experience differ between S and R, but there were also age and experience differences between OS and PS surgeons (Table 1).

**Table 2:** General characteristics

	OS		PS	
	Surgeon %	Resident %	Surgeon %	Resident %
Age				
< 35	8	89	5	82
35-45	34	11	56	18
45-55	30	0	29	0
> 55	29	0	10	0
Experience				
Resident	0	100	0	100
< 5 years	29		34	
5-10 years	15		27	
> 10 years	56		39	

*OS* Orthopedic Surgery, *PS* Plastic Surgery

Age: *n* = 312 (missing value, *n* = 23)

Experience: *n* = 335

### Knowledge of SIC elements

Just over half (OS 51 %, PS 55 %, ns) of the respondents were aware that a competence check of the patient is part of the SIC process (Question 18, Table 3). Almost all responders (93 %) knew that providing information is the second obligatory aspect of SIC. Interestingly, the awareness on the third aspect of SIC (“recording”) was significantly better in the OS group compared to the PS group (*p* = 0.002). Overall, one in four surgeons was not aware that recording of SIC is an essential step (S 74 %, R 91 %, *p* < 0.002, not in table). Moreover, the PS group erroneously thought significantly more often than the OS group that a signature of either patient (*p* = 0.0001) or doctor (*p* = 0.0001) is necessary for adequately recording SIC (Table 3).

**Table 3:** Knowledge of SIC Elements and SIC in daily practice

Question	Answer	Total	OS	PS	*P*-Value
Q 18 What are the elements of SIC?		Y/N% (*n* = 335)	Y/N% (*n* = 251)	Y/N% (*n* = 84)	
	Evaluation of competence	52/48	51/49	55/45	Ns
	Patient education	93/7	93/7	95/5	Ns
	Recording of the consent	78/22	79/21	75/25	0.002
	Patients’ signature is obligatory	42/58	36/64	62/38	0.0001
	Surgeons’ signature is obligatory	42/58	37/63	58/42	0.0001
Q14 Are you informing patients on SIC and patient rights?		Y/N% (*n* = 334)	Y/N% (*n* = 250)	Y/N% (*n* = 84)	
	Y/N	37/63	36/64	39/61	Ns
Q15 Who is mainly informing patients on SIC and patients’ rights?		Y/N% (*n* = 335)	Y/N% (*n* = 251)	Y/N% (*n* = 84)	
	Surgeon	40/60	39/61	41/59	Ns
	Resident	22/78	22/78	23/77	Ns
	Nursing staff	10/90	10/90	8/92	Ns
	Leaflets	13/87	14/86	8/92	Ns
Q5 For which type of surgical procedure a SIC is required?		% (*n* = 334)	% (*n* = 250)	% (*n* = 84)	
	All surgical procedures	37	38	32	Ns
	Elective procedures	31	30	32	Ns
	Depending on the surgeon	32	31	36	Ns
Q7 Which check is used to test patient competence?		% (*n* = 334)	% (*n* = 250)	% (*n* = 84)	
	Own clinical judgement	95	95	95	Ns
	Questionnaire	3	3	2	Ns
	No control	2	2	2	Ns
Q8 Is there a SOP on information that is provided to patients?		% (*n* = 333)	% (*n* = 249)	% (*n* = 84)	
	Y/N	66/34	66/34	69/31	Ns
Q10 Do you inform patients on:		Y/N% (*n* = 335)	Y/N% (*n* = 251)	Y/N% (*n* = 84)	
	the diagnosis and indication ?	99/1	99/1	98/2	Ns
	the surgical procedure ?	97/3	97/3	98/2	Ns
	complications ?	98/2	98/2	96/4	Ns
	alternative treatment options ?	89/11	89/11	91/9	Ns
Q13 Which complication percentage do you us to inform your patients?		Y/N% (*n* = 335)	Y/N% (*n* = 251)	Y/N% (*n* = 84)	
	Rates from literature	66/34	68/32	60/40	Ns
	Rates from own department	31/69	36/64	18/82	0.002
	Personal rates	18/82	17/83	19/81	Ns
Q11 How do you check if the patient has understood the information ?		Y/N% (*n* = 335)	Y/N% (*n* = 251)	Y/N% (*n* = 84)	
	Repeat back method	9/91	9/91	8/920	Ns
Q6 Do you use SIC forms in daily practice ?		Y/N% (*n* = 331)	Y/N% (*n* = 248)	Y/N% (*n* = 83)	
	Y/N	26	21	42	0.001
Q17 Is there a check prior to the surgical procedure if the SIC process is correctly completed?		Y/N% (*n* = 334)	Y/N% (*n* = 250)	Y/N% (*n* = 84)	
	Y/N	36/64	37/63	32/68	Ns
Q9 Which supporting tools are you using?		Y/N% (*n* = 335)	Y/N% (*n* = 253)	Y/N% (*n* = 84)	
	Leaflets	98/2	97/3	99/1	Ns
	Informative movies/DVD	25/75	31/69	6/94	0.05
	Computer software / websites	46/54	50/50	32/68	0.0001
	Nursing staff	58/42	69/31	27/73	0.008
Q22 Are you interested in using SIC software?		Y/N% (*n* = 334)	Y/N% (*n* = 250)	Y/N% (*n* = 84)	
	Y/N	79/21	78/22	82/18	Ns
Q19 Is SIC important for doctors?		Y/N% (*n* = 330)	Y/N% (*n* = 246)	Y/N% (*n* = 84)	
	Y/N	89/11	89/11	86/14	
Q20 Do you think that patients realise the importance of SIC?		Y/N% (*n* = 330)	Y/N% (*n* = 246)	Y/N% (*n* = 84)	
	Y/N	57/43	57/43	54/46	

*Q* Question, *Y* Yes, *N* No, *n* number, *Ns* not significant, *SOP* standard Operating Procedure, *SIC* Surgical Informed Consent

### SIC in daily practice

Only 37 % of the respondents informed their patients on the SIC process itself (Q14). 40 % considered the surgeon responsible for informing patients on SIC. The role for residents regarding this task was considered significantly different by surgeons as compared to residents (R 43 % vs. S 17 %, *p* < 0.0001, not in table). Very few respondents relied on nursing staff or leaflets as a means to inform patients on SIC (Q18).

From a legal standpoint, patients have to give consent to any invasive or operative procedure. Just 37 % of the respondents always asked for consent whereas the remaining did not. 31 % asked for consent only in elective cases and 32 % only in cases where consent was deemed crucial by the doctor (Q5).

### Elements of SIC

#### Assessment of preconditions

Almost all respondents (98 %) checked patient competence. However, very few used questionnaires or checklists (3 %), so the majority relied on their own judgment (95 %) (Q7).

#### Provision of information

Sixty-six percent of the respondents claimed to have a standard operating procedure (SOP) regarding specifics of standard information that was communicated to the patient in the preoperative situation (Q8). There was a high consistency regarding the information given to the patients on diagnosis (99 %), operative procedure (97 %) and complications (98 %). There was no difference between OS and PS in informing their patients about alternative treatment options (OS 89 %, PS 89 %, ns). However, significantly more surgeons (92 %) claimed to inform their patients about alternatives compared to residents (80 %, *p* < 0.005, not in table) (Q10).

An SOP regarding communication of potential complications associated with the procedure was significantly more present in the OS group compared to the PS group (OS 60 % vs. PS 44 %, *p* < 0.01, not in table). Surgeons were more aware of having a SOP on complication information than residents (S 59 % vs. R 44 %, *p* < 0.025, not in table) (Q13).

One of three respondents (34 %) never specified the incidence of a complication, whereas 66 % used complication rates from the literature. 31 % of the surgeons used department rates, and 18 % also provided personal complication rates. Compared to surgeons, residents were far less likely to provide complication rates from their departments (S 31 % vs. R 21 %, *p* = 0.04) or personal rates (S 18 % vs. R 1 %, *p* < 0.001, not in table).

Several methods may be used by a surgeon to verify whether information is understood by his patient. Merely asking if a patient has any questions or asking if everything is understood is not sufficient. The repeat-back method is far more adequate, but this method is seldom used by either group (9 %) (Q11).

#### Stage of Consent

Although SIC forms are not obligatory in the Netherlands, the PS group used them significantly more often compared to the OS group (OS 21 % vs. PS 42 %, *p* = 0.001) (Q6).

An important element of patient safety programs is a preoperative check on whether SIC was followed [9, 12] (Kuo CC and Robb WJ 3rd, 2012, Leclercq WK et al., 2013) (9;12) (9;12) (9;12) (10;13). However, just one of 3 respondents (36 %) claimed to check for an adequate preoperative consent (Q17).

### SIC support tools

Various tools may aid surgeons and residents in facilitating the SIC process. Leaflets containing patient information were frequently used by all parties (98 %). However, other tools such as movies, software programs/websites and nursing staff for informing patients were more often used by the OS group (Q9). Significantly more surgeons than residents used modern tools such as websites to inform patients (OS surgeons 55 % vs. OS residents 27 %, *p* < 0.0001; PS surgeons 39 % vs. PS residents 16 %, *p* = 0.04, not in table).

Seventy-nine percent of the respondents claimed to have an interest in using interactive computer programs for SIC (Q22).

### Medicolegal consequence of present day SIC practice

Most respondents stated that they believed SIC was especially important for medical staff (89 %). In contrast, only 57 % thought that patients were aware of the importance of SIC (Q19). A total of 15 % (OS 12 %, PS 16 %, ns) of the surgeons and 7 % (OS 4 %, PS 9 %, ns) of the residents had received one or more SIC-related official complaint in the preceding five years (Q21, not in table).

## Discussion

A previous study demonstrated that SIC is poorly implemented in the daily practice of Dutch general surgeons [3]. One explanation was a possible high percentage of (semi-)acute procedures in general practice. Conversely, as procedures in the orthopedic and plastic surgical fields are mainly elective, it was hypothesized that the process of SIC would be better implemented in these two groups. However, the knowledge in the OS and PS groups were not better compared to GS (checking for competence OS 51 %, PS 55 %, GS 62 %; recording of consent OS 79 %, PS 75 %, GS 88 %). In daily practice this same observation can be made. To our surprise, the OS and PS asked less often for consent, even in elective cases, compared to GS (OS 38 %, PS 32 %, GS 49 %); moreover, a pre-operative check if consent was adequately provided by the patient was at least equally worse (OS 37 %, PS 32 %, GS 46 %). This same observation can be made in almost all questions asked. There was one exemption: Slightly more respondents in this study reported to have a standard operating procedure (SOP) for SIC in their department (OS 66 %, PS 69 %, GS 61 %). Results of the present study generally indicate that knowledge and daily skills of SIC are also limited in both orthopedic and plastic practices. Moreover, surgeons and residents performed equally poorly.

Most European law countries (including the Scandinavian) have strict laws on patient rights and SIC. Previous studies show low knowledge scores on most issues concerning SIC, and consequently, daily practice is suboptimal [3, 13]. In many studies the use of SOPs, tools and standard forms enhances the quality of patient care [5, 6, 14]. In this study, knowledge was poor and the daily practice results were substandard. Improvement of the SIC process in the orthopedic and plastic surgical field is required.

Solutions to improve the quality of the SIC process are available. Better training for medical staff should enhance the knowledge on SIC and should be implemented in surgical traineeships; introduction of best practice SOPs, adequate tools and standard SIC forms should enhance daily practice. SIC forms can aid medical staff during the SIC process if designed properly [15–17]. In the PS group 42 % already used SIC forms, compared to 21 % of the OS group. The use of interactive online SIC programs might be the next leap forward [3, 9]. Many respondents were interested in using interactive tools to aid the SIC process. We have developed an online SIC program for patients referred for several procedures such as blepharoplasty, basal cell carcinoma of the skin, breast reduction surgery and inguinal hernia repair. These programs will be tested in upcoming trials to test feasibility in daily practice.

The strength of this study is the high response rate from many departments over the country and the opportunity to compare these results with our earlier study in the GS group. Some differences between the OS and PS groups are found, but on the majority of the questions, results are in concordance with this previous study [9].

There are, however, also limitations to this study. SIC is not a very popular topic in the surgical field, and we had trouble getting respondents. The low individual response rate reflects this matter, and selection bias is therefore possible. But if present, this study is likely to provide too optimistic of a view of reality as the results of the respondents were not good, and they might even be lower in the rest of the population who did not respond. Many respondents asked for more training and better SOPs, forms and tools, and hopefully this article will help to improve awareness on this topic and enhance the quality of the SIC process in the future.

## Conclusions

In conclusion, informed consent in surgery is a rapidly developing area in medicine [8]. The role of surgeons in SIC is truly important and determines its quality. The SIC process is, however, complex. Transmitting correct and adequate information to make the patient well-informed is crucial [8]. There are many tools to aid patients: Not solely to inform of surgical complication rates, but also to aid the patient in the decision making process and to reinforce the bond between surgeon and patient [8]. Current developments in SIC are not implemented in daily practice, according to this study, but many opportunities are available to improve the SIC process as wanted by many patients and doctors.

## Additional file

Additional file 1: Questionnaire. (DOC 36 kb)

## Abbreviations

GS: General surgery
NOV: Dutch Scientific Association of Orthopedic Surgeons
NVPC: Dutch Society for Plastic Surgery
OS: Orthopedic Surgery
PS: Plastic Surgery
SIC: Surgical informed consent
SOP: Standard operating procedure
WGBO: Dutch Medical Treatment Contract Act
