# Supplementary material for: Challenging the knowledge base and skillset for providing surgical consent by orthopedic and plastic surgeons in the Netherlands: an identified area of improvement in patient safety
Source: Patient Saf Surg. 2016 Oct 22;10:21. doi: 10.1186/s13037-016-0110-0 (PMC5075159; doi:10.1186/s13037-016-0110-0)
Supplement: Additional file 1: — Questionnaire. (DOC 36 kb) [file 13037_2016_110_MOESM1_ESM.doc]

Appendix A. Questionnaire

1. Name
2. Type of surgical staff (resident, consultant)
3. Type of hospital
4. Does your department have a standard operating procedure (SOP) describing the informed consent process?
5. Which surgical procedures require an informed consent process?
6. In which way is the informed consent process recorded?
7. How do you verify if a patient is competent enough to follow the consecutive steps of an informed consent procedure?
8. Does your department have a SOP regarding quality and quantity of information that is exchanged with a patient during a pre-operative consult?
9. Are any supporting tools used for informing your patients at your outpatient clinic?
10. Which of the following items are discussed with the patient during a pre-operative consult?
11. How do you verify if the patient has indeed understood the information?
12. Which complications do you discuss with your patient?
13. Which complication rates do you mention to your patient?
14. Have your patients received education on the elements of informed consent?
15. If so, who provided the education?
16. Where is the informed consent form stored?
17. Is there a check on a correct informed consent process prior to a surgical procedure?
18. Which elements form an informed consent process?
19. How important is the informed consent process in relation to the total of care that is provided to a patient?
20. According to you, what is your patients opinion on these matters ?
21. Did you have any legal cases in relation to the informed consent process in the previous 5 years ?
22. Are you interested in using any software that may potentially support a surgical informed consent process?
23. Do you have any remarks?
